# Advanced responder training priorities in Kenya: A Delphi study with implications for low- and middle- income countries

**DOI:** 10.1016/j.afjem.2026.100968

**Published:** 2026-03-30

**Authors:** Anagha B. Thiagarajan, Ashwin J. Kulkarni, Simon O. Ogana, Dinnah A. Okwiri, Haleigh Pine, Maxwell Klapow, Mary Falstin, Peter G. Delaney, Zachary J. Eisner, Nathanael J. Smith, John Arudo

**Affiliations:** a University of California, Irvine, School of Medicine, Irvine, CA, USA; b University of Miami, Department of Anesthesiology, Perioperative Medicine and Pain Management, Miami, FL, USA; c Masinde Muliro University of Science and Technology, Nursing Research Education and Management, Kakamega, Kenya; d Perelman School of Medicine, University of Pennsylvania, Philadelphia, CA, USA; e Lay First Responders International, Los Angeles, CA, USA; f Department of Experimental Psychology, University of Oxford, Oxford, UK; g University of Michigan Medical School, Ann Arbor, MI, USA; h Cleveland Clinic Department of Orthopaedic Surgery, OH, USA; i Division of Plastic and Reconstructive Surgery, Keck School of Medicine, Los Angeles CA, USA; j Department of Emergency Medicine, Washington University in St. Louis School of Medicine, St. Louis, MO, USA

**Keywords:** Advanced responder training, Emergency medicine, Prehospital care, Sub-Saharan Africa

## Abstract

**Introduction:**

Low- and middle- income countries account for 90 % of global trauma-related mortality. Organized prehospital emergency medical services and formal first responder systems are often lacking, contributing to this disproportionate injury burden. Lay first responder training programs have demonstrated effectiveness in addressing gaps in prehospital care. However, the curriculum, skills, and resources required to implement a context-appropriate advanced responder program remains unclear.

**Methods:**

A three-round modified Delphi consensus was generated at the Masinde Muliro University of Science and Technology (MMUST) in Western Kenya, including paramedics and paramedic lecturers. The 85-item survey consisted of six demographic items, 30 knowledge items, 30 skills items, and 19 resource items. A total of 79 items were included for consensus evaluation.

**Results:**

A total of 26 participants were included in the Delphi process, with 100 % retention across three rounds. Participants were primarily male (53.8 %) and practicing paramedics (61.5 %), with a median of seven years of experience (IQR: 4.0, 9.0). 33 of 79 items met consensus, defined as greater than 70 % agreement. This included 11 items in knowledge category, 14 items in skills category, and eight items in resources category. Participants prioritized content related to multi-system integration, respiratory and cardiovascular emergencies, and the female reproductive system. Particular emphasis was placed on airway and dehydration management, hemorrhage control, and emergency labor. Medication overdose and gastrointestinal complications were assigned lower priority. Participants identified the need for additional equipment and trained instructors to effectively implement advanced responder training.

**Conclusion:**

This three-round, modified Delphi study in Western Kenya establishes a list of context-appropriate essential knowledge, skills, and resources required for advanced responder training. Consensus was achieved on 33 of 79 items. Similar Delphi-based approaches may help guide the development of advanced responder programs in other resource-limited settings.

## African Relevance

• Many African countries lack formal emergency medical services, and this study provides context-specific guidance for developing an advanced Tier-1 responder curriculum appropriate for resource-limited settings.
• Road traffic injuries disproportionately affect African populations, and the identified skills in this modified Delphi study emphasize early stabilization of common prehospital emergencies seen in these settings.
• The consensus-based priorities align with leading causes of preventable mortality across sub-Saharan Africa, enabling targeted capacity building.
• These findings support African paramedic educators and ministries of health by offering a validated framework for scalable advanced responder training that can strengthen community-level emergency care pathways.
• The study was conducted in Western Kenya with local paramedics and lecturers ensuring that the consensus reflects African frontline realities.

## Introduction

In Africa, 91.3 % of the population lacked access to emergency medical services (EMS) in 2017 [1]. Low- and middle-income countries (LMICs) account for over 90 % of the six million annual global injury-related deaths, with road traffic injury (RTI) deaths occurring at nearly three times the rate as compared to high-income countries (HICs) [2]. In Kenya, pedestrians, cyclists, and motorcycle (boda) drivers comprise 62 % of individuals involved in RTIs [3]. Expanding EMS may address up to 45 % of all deaths and 36 % of total disease burden occurring in LMICs [4].

EMS in HICs typically utilize a Tier-2 model with professional first responders practicing advanced life support (ALS) [5]. However, this model is not feasible in most LMIC settings due to limited infrastructure [6]. In 2004, the World Health Organization (WHO) recommended training lay first responders (LFRs) to address the growing trauma burden in LMICs [[7], [8], [9]]. LFRs, classified as Tier-1 responders, are community members trained in basic first response, distinguishing them from Tier-2 professional EMS personnel in HICs [10]. Several LFR programs across sub-Saharan Africa (SSA) have demonstrated effective knowledge acquisition, retention, and community impact [[10], [11], [12], [13]]. Although it is unclear whether Tier-1 or Tier-2 models are optimal in resource-limited settings, little progress has been made in developing advanced curricula for practicing LFRs [13].

We conducted a modified Delphi study to evaluate essential knowledge and skills, as well as the feasibility of an advanced responder training course. Advanced responder programs implemented in LMICs have demonstrated improved trauma outcomes [14]. Equipping responders with advanced skills such as shock and trauma management, obstetrics, automated external defibrillator (AED) usage, and basic pharmacological intervention, builds on existing LFR training with a focus on hemorrhage control, airway management, fracture immobilization, and patient transport [14]. A framework for a Tier-1 advanced responder training course may draw on ALS and Prehospital Trauma Life Support (PHTLS) principles [15]. To our knowledge, no modified Delphi consensus-based approach has been used to generate an advanced Tier-1 curriculum [16]. This study aims to build on WHO recommendations to advance context-appropriate Tier-1 advanced responder training.

## Methods

The modified Delphi-based study was conducted at Masinde Muliro University of Science and Technology (MMUST) in Kakamega, Kenya. A modified Delphi approach is widely applicable in areas where evidence is limited, and expert opinion is beneficial to establish consensus [16]. Previous Delphi studies include between 15 - 50 individuals for consensus generation [17]. This study followed CREDES (Conducting and Reporting Delphi Studies) guidelines [18].

### Inclusion and exclusion criteria

Paramedics and paramedic lecturers meeting inclusion criteria were identified through the MMUST employee electronic database (Table 1). Paramedics and paramedic lecturers were chosen given their experience with PHTLS and ALS curricula and encountering prehospital trauma. Paramedics were defined as trained prehospital emergency care providers delivering frontline trauma care in resource-limited settings, often with variable training and scope of practice. Experience with PHTLS and ALS curricula was considered appropriate for expert classification because these frameworks emphasize structured trauma assessment, prioritization of life-threatening conditions, and decision-making under resource-limited conditions, core competencies informing advanced prehospital skills suitable for Tier-1 lay responders. A total of 26 paramedic lecturers and paramedics affiliated with MMUST (via MMUST electronic employee database) were randomly sampled from 221 individuals who met inclusion criteria. Random sampling was conducted through a computer-generated random number generator, and 26 unique numbers corresponding to eligible participants were selected without replacement. The individuals associated with these numbers were invited to participate in the Delphi process.

**Table 1:** Delphi participant inclusion and exclusion criteria.

Inclusion Criteria	Exclusion Criteria
18 years or older	Younger than 18 years
Certified paramedic employed by MMUST	Paramedic student
Paramedic lecturer employed by MMUST	Certified paramedic or paramedic lecturer not employed by MMUST
Employed by MMUST for 12 months or longer	Employed by MMUST for <12 months

MMUST=Masinde Muliro University of Science and Technology.

### Study design

This modified Delphi study was performed across three rounds [19]. The first round contained a total of 85 questions, with six demographic questions covering participant identification number, gender, age range, sub-county of residence, occupation (paramedic or paramedic lecturer), and years of experience. This was followed by desired knowledge, skill, and resource items. In the second round, individual responses alongside group averages were returned to the panelists. Participants were then instructed to complete a second questionnaire to assess their level of agreement. This was repeated for the third round. Participants were given an opportunity to add any additional knowledge, skills, and resources they found meaningful through a free-response question at the end of the questionnaire. Consensus was defined as 70 % or more participants selecting “Agree” or “Strongly Agree” on a five-point Likert scale, achieved after three rounds [[20], [21], [22]].

### Questionnaire content

**Fig. 1:**
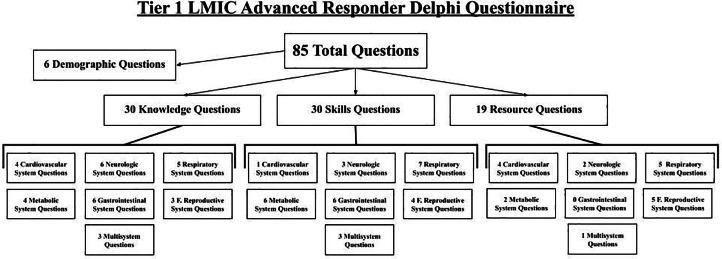
Framework for advanced responder delphi questionnaire.

The Delphi questionnaire was developed by the study team using an iterative process grounded in establish education materials from LFR International [15]. Two investigators reviewed LFR International’s existing PHTLS and ALS training curricula as they comprise a broad range of high-priority prehospital topics previously validated through field implementation, including obstetrics, strokes, seizure, cardiac arrest, dehydration, and multisystem emergencies [15]. The draft questionnaire underwent internal review by a three-member advisory group with expertise in emergency care, prehospital training, and global health education. The Delphi questionnaire included three categories: knowledge, skills, and resources. There were a total of 30 items in the knowledge category; four were multisystem, three focused on the cardiovascular system, six focused on the neurological system, five focused on the respiratory system, four focused on the metabolic system, five focused on the gastrointestinal system, and three focused on the female reproductive system. There were a total of 30 items in the skills category; three questions were multisystem, one focused on the cardiovascular system, three focused on the neurological system, seven focused on the respiratory system, six focused on the metabolic system, six focused on the gastrointestinal system, and four focused on the female reproductive system. There were a total of 19 items in the resources category; two questions were multisystem, three focused on the cardiovascular system, four focused on the neurological system, three focused on the respiratory system, two focused on the metabolic system, five focused on the female reproductive system (Fig. 1)**.**

### Data collection procedures

The first round of the Delphi study was conducted remotely via Qualtrics two weeks prior to the one day in-person intervention. On March 26, 2024, the second and third round of the modified Delphi consensus approach was conducted in-person over the course of two, three-hour sessions at MMUST. The second and third round included participants that successfully completed the first round. Data encompassed responses from all three rounds of the Delphi study. Positive and negative consensus followed stringent criteria, looking for 70 % or more of respondents agreeing or disagreeing with the five-point Likert statements to establish consensus. Items reaching a positive or negative consensus were noted. Consensus items are defined as those established by the third and final round of the modified Delphi study.

Several authors (ABT, AJK, HP, MK, MF, PGD, ZJE, and NJS) are affiliated with LFR International, the organization that funded this study and whose PHTLS and ALS curricula informed development of the Delphi questionnaire. To mitigate potential funder-related bias, the Delphi panel was composed of independent external experts who are not affiliated with LFR International.

### Data analysis

To analyze the first round of responses, we gathered responses from the 85-item questionnaire (with six demographic items). Items receiving positive or negative consensus (defined as 70 % or more of the respondents agreeing or disagreeing) were noted. This was repeated for the second and third round of responses. Items reaching consensus in all three rounds were identified as final consensus items to be included in a Tier 1 advanced responder training program.

Ethical approval was granted by the Republic of Kenya’s National Commission for Science and Technology (NACOSTI/P/24/33,902) and the MMUST Institutional Ethics Review Committee (MMUST/IERC/006/2024).

## Results

Of 221 MMUST-affiliated paramedic lecturers and paramedics invited to participate in this modified Delphi study, 26 were randomly selected to participate. In total, ten (38.5 %) were paramedic lecturers and 16 (61.5 %) were paramedics. Around half of the participants were male (53.8 %), had a median age range of 31–40 years, and a median occupational experience of seven years (IQR: 4.0, 9.0) (Table 2). All 26 individuals participated in all rounds of the Delphi study, yielding a 100 % retention rate.

**Table 2:** Delphi participants’ demographic data.

Demographic Characteristics	N	Percentage (%)
**Gender**		
Male	14	53.8
Female	12	46.2
**Age Range**		
21 - 30	10	38.5
31 - 40	4	15.4
41 - 50	8	30.8
50+	4	15.4
**Occupation**		
Paramedic Lecturer	16	61.5
Paramedic	10	38.5
**Years of Occupation**		
Mean	8.9	
Standard Deviation	5.7	
Median	7.0	
1st Quartile	5.0	
3rd Quartile	9.0	

### Consensus curriculum items

In the first round, participants evaluated 79 proposed curriculum items. These items were subsequently re-rated in two additional rounds, and final consensus was determined after completion of the third round. Following the three Delphi rounds, 33 of the 79 proposed items (42 %) met the predefined consensus threshold for inclusion in the advanced responder training curriculum. Of 33 items meeting consensus, 11 were related to knowledge, 14 to skills, and eight to resources (Table 3). No additional statements were added to the questionnaire by participants.

**Table 3:** Delphi Statement Items Qualifying for Inclusion after Round 3.

	Participants who replied, “Strongly Agree” or “Agree” (%)			Round 1 vs Round 3 p-value
	Round 1	Round 2	Round 3	
*KNOWLEDGE*				
*Cardiovascular System*				
Understanding causes of chest pain should be included in advanced level training.	100	88.5	73.7	0.01
Understanding causes for loss of consciousness, lightheadedness, and syncope (fainting) should be included in advanced level training.	92.3	88.5	80.4	0.12
*Female Reproductive System*				
Understanding pregnancy related emergencies (ie. ectopic pregnancy, spontaneous abortion) should be included in advanced level training.	100	96.2	73.7	0.01
Understanding signs/stages of labor should be included in advanced level training.	92.3	80.8	73.7	0.04
*Gastrointestinal System*				
Understanding the various causes of abdominal pain should be included in advanced level training.	100	92.3	73.1	0.01
*Metabolic System*				
Understanding the causes for dehydration should be included in advanced level training.	96.2	88.5	80.8	0.09
*Multisystem*				
Understanding human anatomy should be included in advanced level training	92.4	88.5	80.4	0.12
Understanding basic organ function and how to identify the main vital organs (heart, brain, kidneys, liver, and lungs) should be included in advanced level training.	100	88.5	73.7	0.01
Understanding the relationship between the various organ systems (ie. respiratory, circulatory, digestive, endocrine, nervous, etc.) should be included in advanced level training.	96.2	92.3	73.7	0.02
*Neurological System*				
Understanding how to identify different levels of consciousness (ie. alert, responds to visual stimuli, responds to painful stimuli, unresponsive) should be included in advanced level training.	96.2	84.6	76.9	0.04
*Respiratory System*				
Understanding causes of shortness of breath should be included in advanced level training.	96.2	96.2	76.9	0.04
*SKILLS*				
*Cardiovascular System*				
Understanding how to perform CPR/Chest compressions should be included in advanced level training.	92.3	100	84.6	0.4
*Female Reproductive System*				
Understanding how to identify pregnancy and/or a pregnant individual should be included in advanced level training.	96.2	88.4	84.6	0.16
Understanding how to identify the signs of labor (ie. contractions) should be included in advanced level training.	92.3	88.4	76.9	0.13
*Metabolic System*				
Understanding the symptoms and patient stabilization of hypoglycemia and the treatments for hypoglycemia should be included in advanced level training.	96.2	92.3	84.6	0.16
*Multisystem*				
Understanding the symptoms and patient stabilization of sepsis (organ failure) should be included in advanced level training.	92.3	84.6	73.7	0.04
Understanding how to stabilize a patient that is unconscious or not fully conscious (semi-unconscious) should be included in advanced level training.	100	96.2	84.6	0.04
Understanding how to place and administer fluids through an IV in patients should be included in advanced level training.	92.3	84.6	76.9	0.13
*Neurological System*				
Understanding the symptoms and patient stabilization for stroke (facial droop, arm drift, speech, time) should be included in advanced level training.	100	88.5	73.7	0.01
Understanding the symptoms and patient stabilization that is actively experiencing a seizure should be included in advanced level training.	92.3	92.5	84.6	0.23
*Respiratory System*				
Understanding the symptoms and patient stabilization for pneumothorax should be included in advanced level training.	92.3	84.6	73.7	0.04
Understanding the symptoms and patient stabilization of flail chest/cracked ribs should be included in advanced level training.	92.3	84.6	73.7	0.04
Understanding symptoms and patient stabilization of an obstructed airway should be included in advanced level training.	96.2	96.2	88.5	0.65
Understanding how to stabilize a patient that is experiencing shortness of breath should be included in advanced level training.	96.2	96.2	84.6	0.16
Understanding symptoms and patient stabilization of asthma (ie. inhaler) should be included in advanced level training.	88.5	88.5	73.1	0.17
*REQUIRED RESOURCES*				
*Cardiovascular System*				
A portable blood pressure monitor (used to assess patients) should be available for advanced first responders.	96.2	92.3	76.9	0.04
*Metabolic System*				
A glucometer (used to check blood glucose levels) should be available for advanced first responders.	100	96.2	80.8	0.04
Glucose gel (used to treat diabetic emergencies) should be available for advanced first responders.	96.2	88.4	76.9	0.02
*Multisystem*				
Clean drinking water (used to treat dehydration) should be available for advanced first responders.	96.2	69.2	88.5	0.31
*Respiratory System*				
A dressing wound (used to treat pneumothorax) should be available for advanced first responders.	92.3	88.5	76.9	0.13
A bulky dressing bandage (used to treat cracked ribs) should be available for advanced first responders.	96.2	84.6	80.8	0.09
Tools to manage an obstructed airway should be available for advanced first responders.	96.2	96.2	76.9	0.04
Asthma inhalers (used to treat asthma attacks) should be available for advanced first responders.	96.2	84.6	88.5	0.31

IV=intravenous line.

Within the knowledge domain, panelists prioritized foundational clinical concepts relevant to advanced prehospital care, including understanding human anatomy, organ system relationships, and recognition of key emergency presentations such as chest pain, syncope, shortness of breath, dehydration, abdominal pain, and pregnancy-related emergencies.

Within the skills domain, participants emphasized practical stabilization skills for time-sensitive emergencies. These included cardiopulmonary resuscitation, management of airway obstruction, pneumothorax recognition, stroke and seizure management, hypoglycemia management, and stabilization of unconscious patients.

Within the resources domain, panelists identified several essential items required to support advanced responder training and implementation, including airway management tools, asthma inhalers, wound dressings for thoracic injuries, blood pressure monitors, glucometers, glucose gel, and access to clean drinking water.

The complete list of consensus items and round-by-round agreement levels are presented in Table 3, with non-consensus items provided in the Supplementary Table.

## Discussion

This modified Delphi resulted in consensus on 33 items for an advanced Tier-1 first responder training. The advanced responder training curriculum incorporates human anatomy and physiology, emergent medical conditions, advanced skills to stabilize patients, and medical equipment into a future curriculum.

### Cardiovascular (CV) system

A total of five out of 12 (44 %) CV system elements met inclusion criteria. CV disease is becoming more prevalent in LMICs, pointing to a crucial need for cardiovascular system focused elements in an advanced first responder training [23]. Concurrently, local awareness of CV disease is low in LMICs. A teaching module on causes of chest pain, understanding causes for syncope, and teaching first responders how to perform CPR is vital [24]. For instance, a study on advanced first responder development in Bangladesh demonstrated that 71 % of patients receiving CPR following a drowning incident survive [25]. Participants asked for portable blood pressure monitors, a tool that has demonstrated a threefold increase in mortality with systolic blood pressures <80 mmHg in prehospital settings [26]. Altogether, participants placed a focus on the CV system, in-line with prior basic first responder training programs finding hemorrhage control to be most frequently utilized [27].

### Female reproductive system

Participants agreed on the importance of advanced training on female reproductive pathologies as five out of 12 (44 %) items met inclusion criteria. Globally, over 300,000 women die annually during childbirth, with 99 % occurring in LMICs [28]. Participants emphasized understanding of causes for abdominal pain, pregnancy, signs and stages of labor, and emergencies such as ectopic pregnancy or spontaneous abortions. These topics are well-established in ALS curricula, with threatened labor as most common [29]. Out-of-hospital births account for 42 % of births in LMICs in SSA or South Asia compared to 2 % in the US, resulting in LMICs accounting for 81 % of neonatal mortality burden [30]. ALS training on post-partum hemorrhage stabilization, including uterine massage and misoprostol use, can significantly improve maternal and fetal outcomes at a low cost [31]. Implementing these items into advanced responder trainings in LMICS may mitigate mortality due to childbirth in resource-limited settings.

### Gastrointestinal (GI) system

The GI system received minimal priority from participants, with one out of 12 (8 %) items meeting inclusion criteria: understanding the causes of abdominal pain. Participants believed GI topics such as understanding appendicitis, cholecystitis, diverticulitis, GI bleeding, four-quadrant organ identification, and nasogastric tube insertion to be too complex for advanced curricula. This differs from the HIC medical standard of care, warranting further investigation examining the need for implementing GI system focused prehospital care [32].

### Metabolic system

A total of four out of 12 (33 %) metabolic system elements met inclusion criteria, with a focus on diabetic emergencies and dehydration. In most settings, including LMICs, dehydration is difficult to diagnose due to complex pathophysiology and varying presentations [33]. Exertional hyperthermia and heat stroke are conditions that must be caught in the prehospital setting, as epidemiological data shows a 93 % association between heat and increased mortality rates in LMICs [34]. Additionally, participants were in support of including management of hypoglycemia as opposed to hyperglycemia and diabetic ketoacidosis (DKA). In the case of hyperglycemia, its exclusion may be due to low perceived urgency, whereas for DKA, the complexity of management and need for hospital-based interventions (e.g., IV fluids, insulin) likely limits the role of first responders [35]. Thus, participants requested a glucometer and glucose gel to respond to these emergencies.

### Multisystem

A total of six out of seven (86 %) multisystem elements met inclusion criteria, particularly human anatomy/physiology education and organ function/systems integration. This is a core part of PHTLS and ALS curricula in HICs, providing responders with organ system knowledge that should be included in advanced first responder courses [36]. While there is minimal literature on clinical outcomes related to anatomical knowledge in the prehospital setting, prior work shows that clinicians with poor anatomy understanding have worse clinical outcomes [37]. Teaching advanced prehospital care providers major organ systems and anatomy can boost confidence in the prehospital setting.

### Neurological system

A total of three out of 11 (27 %) neurological system elements met inclusion criteria, with participants viewing seizures, strokes, and altered mental status (AMS) as beyond advanced first response skill level. Participants agreed on including education on different levels of AMS and patient stabilization with a cervical collar. Patient stabilization for neurological complications in LMICs is important, as over 80 % of the burden of neurological conditions reside in LMICs with only 20 % of patients receiving timely care [38,39]. Furthermore, cervical collars with a rigid backboard have been a mainstay of ALS and PHTLS curricula to prevent secondary spinal cord trauma. Recent studies, however, suggest that low-cost, towel-based methods of cervical spine immobilization may be a feasible alternative in low- and middle-income countries (LMICs) [[40], [41]]. While stroke lesion identification and seizure management were identified as beyond the scope of advanced first responder training, patient stabilization and spinal immobilization should be included as it is frequently cited in basic first responder curriculum in LMICs [10].

### Respiratory system

Participants placed a large emphasis on the respiratory system, with ten out of 17 (58 %) of items meeting inclusion criteria. Importance was placed on asthma, pneumothorax, flail chest, and airway obstruction with a desire for provision of asthma inhalers, dressing wounds, bandages, and an emergency airway kit. Epidemiological studies state that LMICs bear 90 % of the asthma burden with some reporting that over 90 % of individuals lack albuterol treatment [42]. Albuterol is priced at <6 USD per 200 doses in Kenya, a cost-effective advanced first responder item to be included. Non-occlusive dressings and needle thoracostomy decompression remain the mainstay of open pneumothorax stabilization and tension pneumothorax prevention, which can also be included in first aid kits [43]. Airway obstruction is common in elderly patients and has shown to be relieved by the abdominal thrusts in over 86 % of cases, another future advanced responder curriculum element [44]. Flail chest treatment will require hospital-level care, though advanced responders can be taught to apply manual pressure to prevent hypoventilation during transport [45]. Just 31 % of participants advocated for naloxone’s utility in advanced responder first aid kits to reverse opioid-induced respiratory depression, a sharp contrast to HICs where naloxone is a mainstay of EMS and lay-person care reducing mortality to just 0.8 % when administered, justifying its high price tag of 25 USD per dose [46]. Opioid-related fatalities are six-fold higher in the US compared to Kenya at 300 deaths compared to 48 deaths per million, respectively [47]. However, these rates are climbing as 87 % of the world’s illicit seizures due to opioids occur in Africa [48]. As the per-capita overdose mortality rates are growing, naloxone inclusion in the advanced responder curriculum in LMICs warrants further investigation.

### Local capacity

Participants expressed concerns regarding availability of equipment, trained personnel, and institutional support required to implement an advanced responder training course. These perspectives reflect the views of professional prehospital providers and educators and may differ from those of lay first responders (LFRs), community facilitators, or occupational first responders who are often responsible for day-to-day implementation of Tier-1 systems. Nonetheless, participants emphasized that advanced responder training should only be considered in settings where a basic responder program has already been sustainability established. Public institutions may be lobbied to support such programs given the demonstrated benefits of EMS development in Uganda, Sierra Leone, and Rwanda [[49], [50]]. County governments may contribute through local budgets, and international partners can assist with initial program development, though long-term sustainability will require domestic funding and local ownership.

## Limitations

This study has several limitations. Generalizability is limited by the composition of the Delphi panel and study setting, as participants were exclusively paramedics and paramedic lecturers from a single academic institution in Western Kenya. Accordingly, these findings are most directly applicable to the Kenyan prehospital care context, and extrapolation to other LMICs should be interpreted cautiously. In addition, this group was selected for their familiarity with ALS and PHTLS frameworks, though their perspectives may prioritize clinical feasibility over contextual and cultural constraints faced by Tier-1 lay first responders, potentially producing consensus items that are aspirational rather than immediately scalable or sustainable. Though this study followed the validated CREDES framework to ensure methodological rigor, the Delphi approach is based on expert opinion and cannot substitute for real-world validation [18]. Future studies should include heterogeneous panels with active lay responders, community health workers, and occupational first responders, alongside field-based evaluations, to improve ecological validity and confirm practical applicability.

## Conclusions

Our three-round modified Delphi study in Western Kenya demonstrates this approach is feasible to generate a list of curricular knowledge, skills, and resources essential for context-appropriate advanced responder training, yielding consensus on 33 out of 79 items. Participants placed an emphasis on learning related to multi-system integration, the respiratory system, the cardiovascular system, and the female reproductive system with stress placed on airway management, hemorrhage control, dehydration response, and labor. Topics such as medication overdose or gastrointestinal complications received comparatively diminished priority. These findings should help guide future iterations of curriculum generation for advanced prehospital first responder training courses.

## Author contributions

AT: Conceptualization, Investigation, Data Curation, Writing - Original Draft, Writing - Review & Editing, Project Administration; AK: Conceptualization, Data Curation, Writing - Review & Editing, Supervision, Visualization, Project Administration; SO: Conceptualization, Writing - Review & Editing, Supervision, Project Administration; DO: Conceptualization, Writing - Review & Editing, Supervision, Project Administration; HP: Conceptualization, Writing - Review and Editing, Project Administration; MK,MF,PD,ZE,NS,JA: Writing - Review & Editing, Supervision, Project Administration. All authors approved the version to be published and agreed to be accountable for all aspects of the work.

## Funding

LFR International, a 501(c)(3) non-profit organization focused on EMS development in resource-limited settings funded the study.

## Dissemination of results

Study results were communicated to Masinde Muliro University of Science and Technology (MMUST) paramedic lecturers, paramedics, and academic leadership through direct feedback sessions and post-study correspondence. These findings were shared to inform local curriculum development and strengthen the university’s role in advancing prehospital training in Western Kenya.

## Declaration of competing interest

We, the authors declared the following interests: ABT, AJK, HP, MK, MF, PGD, ZJE, and NJS are affiliated with LFR International, the organization that funded this study and whose PHTLS and ALS curricula informed development of the Delphi questionnaire.

## Appendix Supplementary materials

Image, application 1

## Footnotes

Supplementary material associated with this article can be found, in the online version, at doi:10.1016/j.afjem.2026.100968.
