# Supplementary material for: Advanced responder training priorities in Kenya: A Delphi study with implications for low- and middle- income countries
Source: Afr J Emerg Med. 2026 Mar 30;16(2):100968. doi: 10.1016/j.afjem.2026.100968 (PMC13068567; doi:10.1016/j.afjem.2026.100968)
Supplement: Supplementary file 1 [file mmc1.docx]

**Supplementary Table 2: Comprehensive Delphi survey results**

| **Body System** | **Question Category** | **Question** | **Percentage of respondents who replied "Strongly Agree" or "Agree"** | | | **Round 1 vs Round 3: p-value** |
| --- | --- | --- | --- | --- | --- | --- |
|  |  |  | **Round 1** | **Round 2** | **Round 3** |  |
| Cardiovascular | Knowledge & Understanding | Understanding causes of chest pain should be included in advanced level training. | 100 | 88.5 | 73.7 | 0.002 |
| Cardiovascular | Knowledge & Understanding | Understanding the various types of chest pain (sharp, stabbing, dull, heavy) should be included in advanced level training. | 96.2 | 65.4 | 69.2 | 0.004 |
| Cardiovascular | Knowledge & Understanding | Understanding causes for loss of consciousness/lightheadedness/syncope (fainting) should be included in advanced level training. | 92.3 | 88.5 | 80.4 | 0.12 |
| Cardiovascular | Symptom Identification & Management | Understanding how to perform CPR/Chest compressions should be included in advanced level training. | 92.3 | 100 | 84.6 | 0.4 |
| Cardiovascular | Tools & Resources | A Holter monitor (used to measure heart rhythms) should be available for advanced first responders. | 92.3 | 84.6 | 26.9 | 0.001 |
| Cardiovascular | Tools & Resources | A defibrillator (used to treat cardiac arrest or heart attacks) should be available for advanced first responders. | 88.5 | 84.6 | 57.7 | 0.02 |
| Cardiovascular | Tools & Resources | A portable blood pressure monitor (used to assess patients) should be available for advanced first responders. | 96.2 | 92.3 | 76.9 | 0.04 |
| Cardiovascular | Knowledge & Understanding | Understanding the stages of pregnancy should be included in advanced level training. | 100 | 92.3 | 57.7 | 0.001 |
| Cardiovascular | Tools & Resources | A printout of the American Heart Association CPR and First Aid Algorithm should be available for advanced first responders. | 100 | 92.3 | 61.5 | 0.001 |
| Female Reproductive | Knowledge & Understanding | Understanding pregnancy related emergencies (ie. ectopic pregnancy, spontaneous abortion) should be included in advanced level training. | 100 | 96.2 | 73.7 | 0.002 |
| Female Reproductive | Knowledge & Understanding | Understanding signs/stages of labor should be included in advanced level training. | 92.3 | 80.8 | 73.7 | 0.04 |
| Female Reproductive | Symptom Identification & Management | Understanding how to identify pregnancy and/or a pregnant individual should be included in advanced level training. | 96.2 | 88.4 | 84.6 | 0.16 |
| Female Reproductive | Symptom Identification & Management | Understanding how to identify the signs of labor (ie. water breaking/contractions) should be included in advanced level training. | 92.3 | 88.4 | 76.9 | 0.13 |
| Female Reproductive | Symptom Identification & Management | Understanding how to identify potential signs of delivery complications (ie. breech delivery, limb presentation, neonatal resuscitations) should be included in advanced level training. | 96.2 | 88.4 | 53.9 | 0.001 |
| Female Reproductive | Symptom Identification & Management | Understanding symptoms and patient stabilization of ectopic pregnancy/spontaneous abortion should be included in advanced level training. | 96.2 | 96.2 | 57.7 | 0.002 |
| Female Reproductive | Tools & Resources | A printout of the normal delivery timeline (used to assist in spontaneous births) should be available for advanced first responders. | 92.3 | 92.3 | 42.3 | 0.001 |
| Female Reproductive | Tools & Resources | A suction bulb (used to assist a baby in breathing after delivery) should be available for advanced first responders. | 96.2 | 96.2 | 65.4 | 0.01 |
| Female Reproductive | Tools & Resources | A kit (clamp, scissors) to assist in cutting the clamping the umbilical cord should be available for advanced first responders. | 96.2 | 84.6 | 65.4 | 0.01 |
| Female Reproductive | Tools & Resources | A printout of APGAR scores (used to assess baby's health at 1 and 5 minutes) should be available for advanced first responders. | 96.2 | 88.5 | 53.8 | 0.002 |
| Female Reproductive | Tools & Resources | Magnesium Sulfate (used to prevent seizures, stopping premature labor, and protecting baby's brain) should be available for advanced first responders. | 96.2 | 88.5 | 26.9 | 0.001 |
| Gastrointestinal | Knowledge & Understanding | Understanding the causes of appendicitis should be included in advanced level training. | 96.2 | 61.5 | 26.9 | 0.001 |
| Gastrointestinal | Knowledge & Understanding | Understanding the causes of cholecystitis should be included in advanced level training. | 88.5 | 57.7 | 26.9 | 0.001 |
| Gastrointestinal | Knowledge & Understanding | Understanding the causes of diverticulitis should be included in advanced level training. | 88.5 | 69.2 | 23.1 | 0.001 |
| Gastrointestinal | Knowledge & Understanding | Understanding the causes of gastrointestinal bleeding and/or abdominal aortic aneurysm should be included in advanced level training. | 92.3 | 88.5 | 42.3 | 0.001 |
| Gastrointestinal | Symptom Identification & Management | Understanding how to perform a nasogastric tube (NGT) insertion should be included in advanced level training. | 92.3 | 84.6 | 26.9 | 0.001 |
| Gastrointestinal | Symptom Identification & Management | Understanding how to identify the organs in the four quadrants of the abdominal space by palpating the patient (Right Upper - Liver, Gallbladder, Left Upper - Stomach, Spleen, Pancress, Right Lower - Appendix, Ovaries, Left Lower - Fallopian Tubes) should be included in advanced level training. | 96.2 | 92.3 | 57.7 | 0.002 |
| Gastrointestinal | Symptom Identification & Management | Understanding symptoms and patient stabilization of a patient with appendicitis should be included in advanced level training. | 96.2 | 76.7 | 26.9 | 0.001 |
| Gastrointestinal | Symptom Identification & Management | Understanding symptoms and patient stabilization of a patient with cholecystitis should be included in advanced level training. | 92.3 | 65.4 | 23.1 | 0.001 |
| Gastrointestinal | Symptom Identification & Management | Understanding symptoms and patient stabilization of a patient with diverticulitis should be included in advanced level training. | 96.2 | 61.6 | 19.2 | 0.001 |
| Gastrointestinal | Symptom Identification & Management | Understanding symptoms and patient stabilization of a patient with gastrointestinal bleeding and/or abdominal aortic aneurysm should be included in advanced level training. | 92.3 | 88.5 | 57.7 | 0.003 |
| Gastrointestinal | Knowledge & Understanding | Understanding metabolism and energy production should be included in advanced level training. | 88.5 | 61.5 | 30.8 | 0.001 |
| Gastrointestinal + Female Reproductive | Knowledge & Understanding | Understanding the various causes of abdominal pain should be included in advanced level training. | 100 | 92.3 | 73.1 | 0.002 |
| Metabolic | Knowledge & Understanding | Understanding diabetes should be included in advanced level training. | 96.2 | 88.5 | 61.5 | 0.002 |
| Metabolic | Knowledge & Understanding | Understanding the causes of diabetic or glucose-related emergencies (hypoglycemia, hyperglycemia, insulin shock, diabetic ketoacidosis) should be included in advanced level training. | 100 | 92.3 | 65.4 | 0.001 |
| Metabolic | Knowledge & Understanding | Understanding the causes for dehydration should be included in advanced level training. | 96.2 | 88.5 | 80.8 | 0.09 |
| Metabolic | Symptom Identification & Management | Understanding the crucial energy sources (fat, protein, sugars) and how they impact metabolism and energy production should be included in advanced level training. | 92.3 | 57.7 | 26.9 | 0.001 |
| Metabolic | Symptom Identification & Management | Understanding normal glucose regulation should be included in advanced level training. | 96.2 | 80.4 | 30.8 | 0.001 |
| Metabolic | Symptom Identification & Management | Understanding the symptoms and patient stabilization of hypoglycemia and the treatments for hypoglycemia should be included in advanced level training. | 96.2 | 92.3 | 84.6 | 0.16 |
| Metabolic | Symptom Identification & Management | Understanding the symptoms and patient stabilization of hyperglycemia and the treatments for hyperglycemia should be included in advanced level training. | 96.2 | 92.3 | 65.4 | 0.004 |
| Metabolic | Symptom Identification & Management | Understanding symptoms and patient stabilization of insulin shock and the treatments for insulin shock should be included in advanced level training. | 100 | 88.5 | 65.4 | 0.001 |
| Metabolic | Symptom Identification & Management | Understanding symptoms and patient stabilization of diabetic ketoacidosis should be included in advanced level training. | 96.2 | 84.6 | 43.3 | 0.001 |
| Metabolic | Tools & Resources | A glucometer (used to check blood glucose levels) should be available for advanced first responders. | 100 | 96.2 | 80.8 | 0.04 |
| Metabolic | Tools & Resources | Glucose gel (used to treat diabetic emergencies) should be available for advanced first responders. | 96.2 | 88.4 | 76.9 | 0.02 |
| Metabolic | Knowledge & Understanding | Understanding human anatomy should be included in advanced level training | 92.4 | 88.5 | 80.4 | 0.12 |
| Multisystem | Knowledge & Understanding | Understanding basic organ function and how to identify the main vital organs (heart, brain, kidneys, liver, and lungs) should be included in advanced level training. | 100 | 88.5 | 73.7 | 0.001 |
| Multisystem | Knowledge & Understanding | Understanding the relationship between the various organ systems (ie. respiratory, circulatory, digestive, endocrine, nervous, etc.) should be included in advanced level training. | 96.2 | 92.3 | 73.7 | 0.02 |
| Multisystem | Knowledge & Understanding | Understanding different types of SHOCK (ie. cardiogenic, hypovolemic, septic, distributive) should be included in advanced level training. | 92.3 | 88.5 | 50 | 0.001 |
| Multisystem | Symptom Identification & Management | Understanding the symptoms and patient stabilization of SEPSIS (organ failure) should be included in advanced level training. | 92.3 | 84.6 | 73.7 | 0.04 |
| Multisystem | Symptom Identification & Management | Understanding how to stabilize a patient that is unconscious or not fully conscious (semi-unconscious) should be included in advanced level training. | 100 | 96.2 | 84.6 | 0.04 |
| Multisystem | Symptom Identification & Management | Understanding how to place and administer fluids through an IV (Intravenous Line) in patients should be included in advanced level training. | 92.3 | 84.6 | 76.9 | 0.13 |
| Multisystem | Tools & Resources | Clean drinking water (used to treat dehydration) should be available for advanced first responders. | 96.2 | 69.2 | 88.5 | 0.31 |
| Neurological | Knowledge & Understanding | Understanding how to identify different levels of consciousness (ie. alert, responds to visual stimuli, responds to painful stimuli, unresponsive) should be included in advanced level training. | 96.2 | 84.6 | 76.9 | 0.04 |
| Neurological | Knowledge & Understanding | Understanding causes of stroke should be included in advanced level training. | 100 | 92.3 | 53.8 | 0.001 |
| Neurological | Knowledge & Understanding | Understanding causes and types of seizures (generalized, absence, partial, status epilepticus) should be included in advanced level training. | 92.3 | 84.6 | 46.2 | 0.002 |
| Neurological | Knowledge & Understanding | Understanding the different phases of seizures (aura, tonic, tonic-clonic, postictal) should be included in advanced level training. | 92.3 | 73.8 | 46.2 | 0.002 |
| Neurological | Knowledge & Understanding | Understanding the various causes of numbness/tingling should be included in advanced level training. | 88.5 | 76.7 | 42.3 | 0.001 |
| Neurological | Knowledge & Understanding | Understanding the causes of spinal cord injuries should be included in advanced level training. | 96.2 | 92.3 | 57.7 | 0.001 |
| Neurological | Symptom Identification & Management | Understanding the symptoms and patient stabilization for stroke (Facial Drop, Arm Drift, Speech, Time) should be included in advanced level training. | 100 | 88.5 | 73.7 | 0.002 |
| Neurological | Symptom Identification & Management | Understanding the symptoms and patient stabilization that is actively experiencing a seizure should be included in advanced level training. | 92.3 | 92.5 | 84.6 | 0.23 |
| Neurological | Symptom Identification & Management | Understanding how to conduct a neurological assessment on a patient that may have had a stroke, or seizure should be included in advanced level training. | 96.2 | 84.6 | 42.3 | 0.001 |
| Neurological | Tools & Resources | A printout of the Cincinnati Prehospital Stroke Scale (used to appropriately identify stroke) should be available for advanced first responders. | 88.5 | 76.7 | 42.3 | 0.001 |
| Neurological | Tools & Resources | A printout of the Glasgow Coma Scale (used to assess level of consciousness) should be available for advanced first responders. | 96.2 | 92.3 | 46.2 | 0.001 |
| Respiratory | Tools & Resources | Naloxone (used to treat drug) should be available to advanced first responders. | 96.2 | 92.3 | 30.8 | 0.001 |
| Respiratory | Tools & Resources | Asthma inhalers (used to treat asthma attacks) should be available for advanced first responders. | 96.2 | 84.6 | 88.5 | 0.31 |
| Respiratory | Knowledge & Understanding | Understanding the causes of pneumothorax (collapsed lung) should be included in advanced level training. | 92.3 | 80.8 | 53.8 | 0.001 |
| Respiratory | Knowledge & Understanding | Understanding the causes of flail chest (cracked ribs) should be included in advanced level training. | 92.3 | 88.5 | 50 | 0.001 |
| Respiratory | Knowledge & Understanding | Understanding the mechanism of breathing (ventilation and exhalation) should be included in advanced level training. | 100 | 84.6 | 61.5 | 0.001 |
| Respiratory | Knowledge & Understanding | Understanding causes of shortness of breath should be included in advanced level training. | 96.2 | 96.2 | 76.9 | 0.04 |
| Respiratory | Knowledge & Understanding | Understanding causes of asthma should be included in advanced level training. | 96.2 | 88.5 | 42.3 | 0.001 |
| Respiratory | Symptom Identification & Management | Understanding the symptoms and patient stabilization for pneumothorax should be included in advanced level training. | 92.3 | 84.6 | 73.7 | 0.04 |
| Respiratory | Symptom Identification & Management | Understanding the symptoms and patient stabilization of flail chest/cracked ribs should be included in advanced level training. | 92.3 | 84.6 | 73.7 | 0.04 |
| Respiratory | Symptom Identification & Management | Understanding symptoms and patient stabilization of an obstructed airway should be included in advanced level training. | 96.2 | 96.2 | 88.5 | 0.65 |
| Respiratory | Symptom Identification & Management | Understanding how to perform an intubation/endotracheal intubation should be included in advanced level training. | 88.5 | 88.5 | 69.2 | 0.09 |
| Respiratory | Symptom Identification & Management | Understanding how to perform a cricothyrotomy should be included in advanced level training. | 88.5 | 80.4 | 46.2 | 0.001 |
| Respiratory | Symptom Identification & Management | Understanding how to stabilize a patient that is experiencing shortness of breath should be included in advanced level training. | 96.2 | 96.2 | 84.6 | 0.16 |
| Respiratory | Symptom Identification & Management | Understanding symptoms and patient stabilization of asthma (ie. metered dose inhaler) should be included in advanced level training. | 88.5 | 88.5 | 73.1 | 0.17 |
| Respiratory | Tools & Resources | A dressing wound (used to treat pneumothorax (collapsed lung)) should be available for advanced first responders. | 92.3 | 88.5 | 76.9 | 0.13 |
| Respiratory | Tools & Resources | A bulky dressing bandage (used to treat flail chest/cracked ribs) should be available for advanced first responders. | 96.2 | 84.6 | 80.8 | 0.09 |
| Respiratory | Tools & Resources | Tools to manage an obstructed airway should be available for advanced first responders. | 96.2 | 96.2 | 76.9 | 0.04 |
| N/A | Local Capacity | Currently, my institution has adeuqate medical equipment and resources to successfully implement an advanced first responder training course. | 61.5 | 15.3 | 23.7 | 0.004 |
| N/A | Local Capacity | Currently, my institution has enough trainers/medical professionals willing to dedicate time and resources to successfully implement an advanced first responder training course. | 76.9 | 46.2 | 30.7 | 0.005 |
